# Morphological characteristics of cerebellum, pons and thalamus in Reccurent isolated sleep paralysis – A pilot study

**DOI:** 10.3389/fnana.2024.1396829

**Published:** 2024-06-19

**Authors:** Eva Miletínová, Monika Kliková, Amálie Dostalíková, Jitka Bušková

**Affiliations:** ^1^National Institute of Mental Health, Klecany, Czechia; ^2^Third Faculty of Medicine, Charles University, Praha, Czechia

**Keywords:** sleep, recurrent isolated sleep paralysis, cerebellum, midbrain, pons, thalamus

## Abstract

**Introduction:**

Recurrent isolated sleep paralysis (RISP) is a rapid eye movement sleep (REM) parasomnia, characterized by the loss of voluntary movements upon sleep onset and/or awakening with preserved consciousness. Evidence suggests microstructural changes of sleep in RISP, although the mechanism of this difference has not been clarified yet. Our research aims to identify potential morphological changes in the brain that can reflect these regulations.

**Materials and methods:**

We recruited 10 participants with RISP (8 women; mean age 24.7 years; SD 2.4) and 10 healthy control subjects (w/o RISP; 3 women; mean age 26.3 years; SD 3.7). They underwent video-polysomnography (vPSG) and sleep macrostructure was analyzed. After that participants underwent magnetic resonance imaging (MRI) of the brain. We focused on 2-dimensional measurements of cerebellum, pons and thalamus. Statistical analysis was done in SPSS program. After analysis for normality we performed *Mann–Whitney U* test to compare our data.

**Results:**

We did not find any statistically significant difference in sleep macrostructure between patients with and w/o RISP. No evidence of other sleep disturbances was found. 2-dimensional MRI measurements revealed statistically significant increase in cerebellar vermis height (*p* = 0.044) and antero-posterior diameter of midbrain-pons junction (*p* = 0.018) in RISP compared to w/o RISP.

**Discussion:**

Our results suggest increase in size of cerebellum and midbrain-pons junction in RISP. This enlargement could be a sign of an over-compensatory mechanism to otherwise dysfunctional regulatory pathways. Further research should be done to measure these differences in time and with closer respect to the frequency of RISP episodes.

## 1 Introduction

Recurrent isolated sleep paralysis (RISP) is a type of sleep disorder characterized by the loss of voluntary movements upon sleep onset and/or awakening with preserved consciousness and preserved function of respiratory and eye movements muscles (American Academy of Sleep Medicine, 2014). These episodes are often accompanied by hallucinations and are perceived as unpleasant (Sharpless et al., 2010). It is a type of parasomnia, which occurs during rapid eye movement (REM) stage of sleep and the episodes can occur repeatedly throughout one night, consecutive nights, during daytime naps and even after long intervals without the episodes (American Academy of Sleep Medicine, 2014). About 40% of general population experiences sleep paralysis at least once in lifetime, yet the recurrence is much less common (Stefani and Hogl, 2021). This phenomenon arises probably from disturbed regulatory mechanisms during transition between REM sleep and wakefulness (Brooks and Peever, 2012). RISP is also often associated with psychological phenomena, such as anxiety (Sharpless, 2016; Sharma et al., 2023). Historically, it has had many culturally specific interpretations. Due to its frightening and rather mysterious nature, a patient experiencing sleep paralysis has been interpreted as being bewitched, frozen, enveloped by a ghost among many other supernatural theories (Dahlitz and Parkes, 1993; Golzari and Ghabili, 2013; Olunu et al., 2018).

Pathophysiological mechanisms in RISP are not fully cleared yet. Potential risk factors include genetic predisposition concerning circadian genes (Stefani and Hogl, 2021), presence of other sleep disorders, psychiatric disorders and/or familial predispositions (Dahlitz and Parkes, 1993; Liskova et al., 2016). Several mechanisms have been described as important in sleep paralysis. Based on the otherwise rare polysomnographic findings in RISP, a combination of wake-like and REM sleep-like brain activity is found in RISP (Hobson, 2009; Terzaghi et al., 2012). So far, there is no clear evidence of macrostructural changes of sleep in RISP, yet the findings are inconsistent (Hsieh et al., 2010). Although there is an inconsistency in macrostructural findings, we have some evidence of microstructural changes in sleep (Klikova et al., 2021). Klikova et al. (2021) describe power spectral analysis of REM sleep in RISP and find higher bifrontal beta activity in this stage of sleep, even though no macrostructural changes were found. This could itself suggest differences in regulatory mechanisms involved in the development of RISP. To our knowledge, the exact nature of changes in sleep–wake regulations leading to RISP has not been clarified. As SP episodes arise from REM sleep, it is a possibility that signaling pathways are disrupted at the level of midbrain and/or pons, as sublaterodorsal nucleus (SLD) and other pontine structures are key players in REM sleep regulation (Herice et al., 2019). Newly also cerebellum has been identified as playing a role in sleep regulation in both health and disease, more specifically idiopathic REM sleep behavior disorder (Canto et al., 2017; Zhang et al., 2020; Lee et al., 2023). Because most of sleep regulatory processes involve thalamus (Gent et al., 2018; Simor et al., 2021), it should also be included into candidate structures playing role in the development of RISP.

The evidence therefore suggests some disturbance in signaling pathways controlling REM sleep, wakefulness, and their transition. Taking that into consideration, our main aim was to discover, whether there might be any morphological differences in size of some brain structures, namely cerebellum, pons and thalamus, that might differ between individuals with RISP and healthy controls (individuals who did not suffer from RISP and/or any other sleep disorder). To do that, we used standardized magnetic resonance imaging (MRI) scans and two-dimensional standardized views (horizontal, sagittal, vertical). We chose these methods as they are widely available to clinicians and the tools are routinely used. We believe this could eventually bring out results relevant to clinical settings, if found useful. In the attempt to exclude any other confounders, such as potential sleep disorders, we performed video-polysomnography of all subjects.

## 2 Materials and methods

### 2.1 Participants

All the patients with RISP were recruited upon answering the online advertisement which was displayed on the web site of National Institute of Mental Health, Klecany, Czech Republic. These participants first underwent the Fearful Isolated Sleep Paralysis interview with a specialized psychologist, then an additional interview with a professional somnologist who determined the nature of the sleep episodes experienced (Klikova et al., 2021). From the original sample (the questionnaires were given to 112 participants), 20 individuals attended further examination in the sleep lab. In the lab, participants underwent another detailed examination led by experienced clinician in order to obtain all the clinically relevant information. From these participants, only 10 agreed to undergo MRI examination and thus were included into the study. Healthy control subjects were given a screening questionnaire to exclude those who did not match the criteria. The main exclusion criteria for both RISP and the control subjects (w/o RISP) groups were the following: (1) any comorbid sleep disorder that might interrupt sleep, such as sleep apnea, periodic leg movements in sleep, restless leg syndrome; (2) potential neurological disorder, such as epilepsy; (3) potential psychiatric disorder that could prevent the participants to complete the study such as severe depression and /or anxiety, sever psychotic disorder or others; (4) claustrophobia and/or nyctophobia that might interfere with MRI examination; (5) Presence of ferromagnetic material, such as metal implants in the body, ferromagnetic tattoos and/or any other object in or on the body, which would prevent him/her to enter the MRI machine; (6) Pregnancy. Healthy control subjects firstly filled the screening questionnaire designed by our lab to gather basic information about subjects. Most of the participants that filled in our questionnaire were university students. We immediately excluded those who worked in shift work, were taking any medication that could possibly affect sleep, such as Z-drugs, Beta-blockers, psychoactive medication, hypnotics, and/or suffer from any serious internal, psychological and/or neurological disorder that would affect their potential participation in the study. Those who passed this part of the selection process underwent the interview with physician, along with analysis of sleep diary filled 2 weeks prior to the interview. All the selected individuals claimed to have regular sleep–wake pattern. The interview conducted by trained somnologist focused on potential sleep and/or any other somatic or psychological disorders. Based on that, we recruited 12 individuals age-matched to our RISP group.

Eventually, after all the recruitment process was finished, we recruited 10 participants with RISP (8 women; mean age 24.7 years; SD 2.4) and 10 healthy subjects (w/o RISP; 3 women, 7 men; mean age 26.3 years; SD 3.7).

### 2.2 Examination procedure

All the individuals underwent video-polysomnography (vPSG) to measure macrostructural sleep parameters and objectively exclude other possible sleep disorders. The detailed description of the process was provided in the work of Klikova et al. (2021). After passing the vPSG evaluation, participants underwent MRI scanning. This was usually scheduled for another appointment the participants left the sleep lab after vPSG and were summoned again depending on their MRI appointment. Before the second appointment participants’ medical condition was checked again in order to assure the inclusion criteria are still met. The distance between vPSG and MRI examination was not longer than 1 week. Participants were given an informed consent related to MRI imaging. Upon signature, they were escorted to the lab where the MRI examination took place. The trained technician provided the basic information about the examination process and also supervised the positioning of the individual into the scanner. The examination itself took approximately 20 min. An MR device with a static magnetic field size of 3 T from Siemens, model Siemens Magneton Prisma, was used for the measurement. The standardized scans were taken in a standard sequence, such as T1-weighted and T2-weighted scans and Flair. The scans were afterwards assessed by neuro-radiologist to make sure no pathological findings such as tumor, cyst, pathological Research Topic of liquid and/or any other life-threatening process was present. This applies to all of our participants.

After collecting all the data, we first analyzed vPSG data. The method of analysis is more in depth described by Klikova et al. (2021). We specifically looked into macrostructural parameters of sleep, such as sleep efficacy (SE), sleep onset latency (SOL), total sleep time (TST), percentage of non-rapid eye movement sleep stage 1 (NREM 1), stage 2 (NREM 2), stage 3 (NREM 3) and REM sleep. We also looked for signs of potential sleep disturbances, such as apnoe/hypopnoea index (AHI), oxygen desaturation index (ODI) and periodic leg movement index (PLMI).

**Figure 1:**
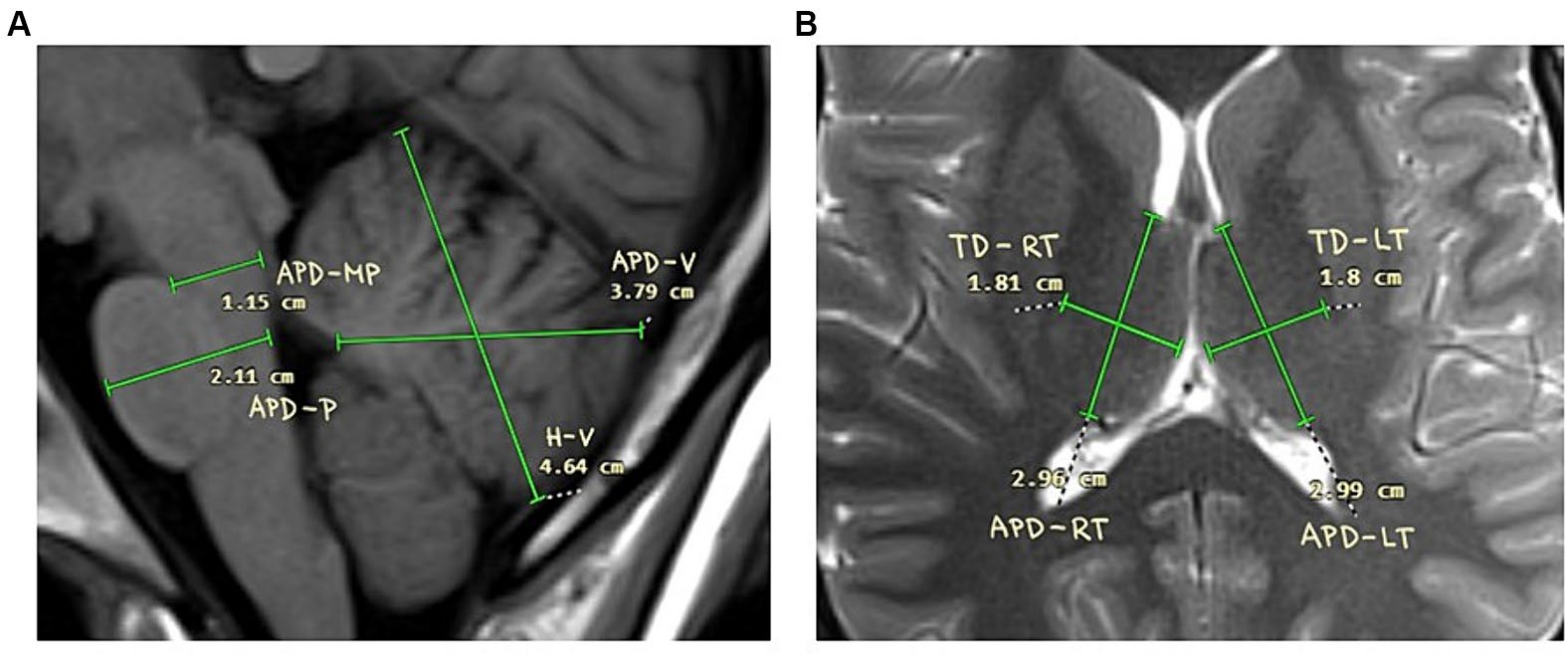
Schemes of the measurements. **(A)** Cerebellum and pontine regions. Height of cerebellar vermis (H-V), antero-posterior diameter of vermis cerebelli (APD-V); antero-posterior diameter of midbrain-pons junction (APD-MP); Antero-posterior diameter of mid-pons (APD-P); Antero-posterior diameter of left thalamus (APD-LT); **(B)** Thalamus. Transversal diameter of left thalamus (TD-LT); Antero-posterior diameter of right thalamus (APD-RT); transversal diameter of right thalamus (TD-RT).

Magnetic resonance imaging was used to measure two dimensional parameters of cerebellum, pons and thalamus. The method of examination was based on previously published techniques (Mohammadi et al., 2008; Jandeaux et al., 2019; Metwally et al., 2021). We measured height of cerebellar vermis (H-V), antero-posterior diameter of vermis cerebelli (APD-V); antero-posterior diameter of midbrain-pons junction (APD-MP); antero-posterior diameter of mid-pons (APD-P); antero-posterior diameter of left thalamus (APD-LT); transversal diameter of left thalamus (TD-LT); antero-posterior diameter of right thalamus (APD-RT); transversal diameter of right thalamus (TD-RT). The illustration of the measurement is available in Figure 1.

### 2.3 Statistical evaluation

All our data was first analyzed for normality of distribution by using *Shapiro–Wilk* test. Based on the results we concluded that the distribution was skewed and thus we used *Mann–Whitney U* test to compare RISP patients and controls.

### 2.4 Ethical statement

This study was approved by the research ethic committee of the National Institute of Mental Health. Written informed consent was obtained from the participants. Participants did not receive any financial compensation for taking part in this experiment.

## 3 Results

### 3.1 Polysomnographic findings

We did not find any statistically significant difference between RISP patients and controls in terms of macrostructural sleep parameters or the scores in AHI, ODI and PLMS. The detailed results are presented in Table 1.

**Table 1:** Objective macrostructural measurement of sleep in RISP and healthy individuals.

	RISP patients Mean (SD)	w/o RISP Mean (SD)	*p* value
Sleep Effectivity (SE)	92.03% (7.45)	87.5% (10.2)	n.s.
Total Sleep Time (TST)	407.8 min (50.2)	381.2 min (42.2)	n.s.
Sleep Onset Latency (SLAT)	32.5 min (11.0)	29.7 min (18.1)	n.s.
REM Sleep Latency	107.6 min (20.1)	79.3 min (8.08)	n.s.
Wake	10% (10.2)	12% (11.4)	n.s.
NREM 1	4.9% (2.8)	6.2% (1.9)	n.s.
NREM 2	46% (5.6)	44% (10.4)	n.s.
NREM 3	21.6% (5.3)	24.4% (9.1)	n.s.
REM	21.6% (5.5)	18.7 (2.8)	n.s.
AHI	4.2 (1.7)	3.5 (0.8)	n.s.
PLMI	1.5 (0.7)	1.2 (0.3)	n.s.

REM, Rapid eye movement sleep; NREM 1, Non rapid eye movement sleep stage 1. NREM 2, Non-rapid eye movement sleep stage 2; NREM 3, Non rapid eye movement sleep stage 3; AHI, Apnoe hypopneo index; PLMI, periodic leg movement in sleep index.

### 3.2 Neuroimaging

**Figure 2:**
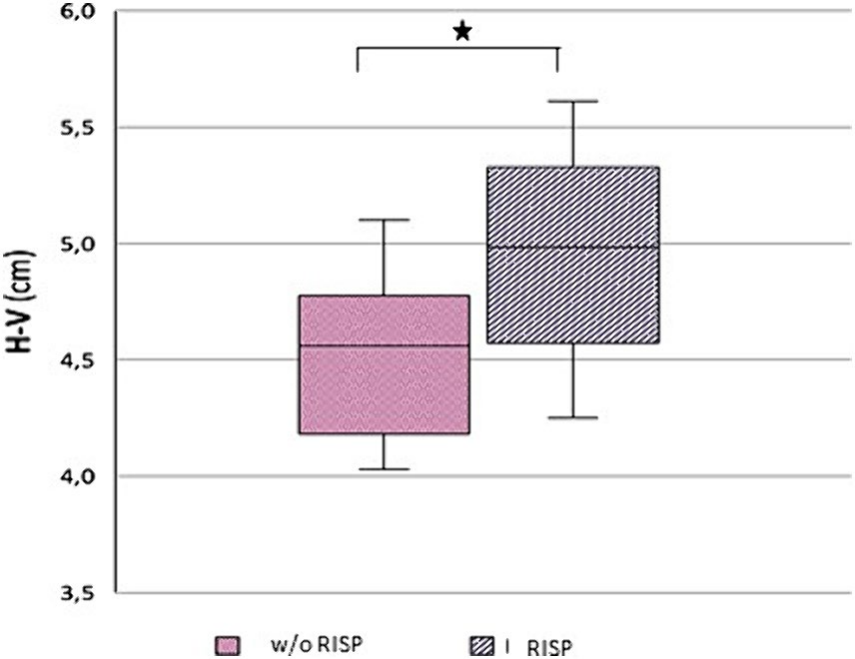
Height of Vermis (H-V) in w/o RISP (pink) compared to RISP patients (gray).

**Figure 3:**
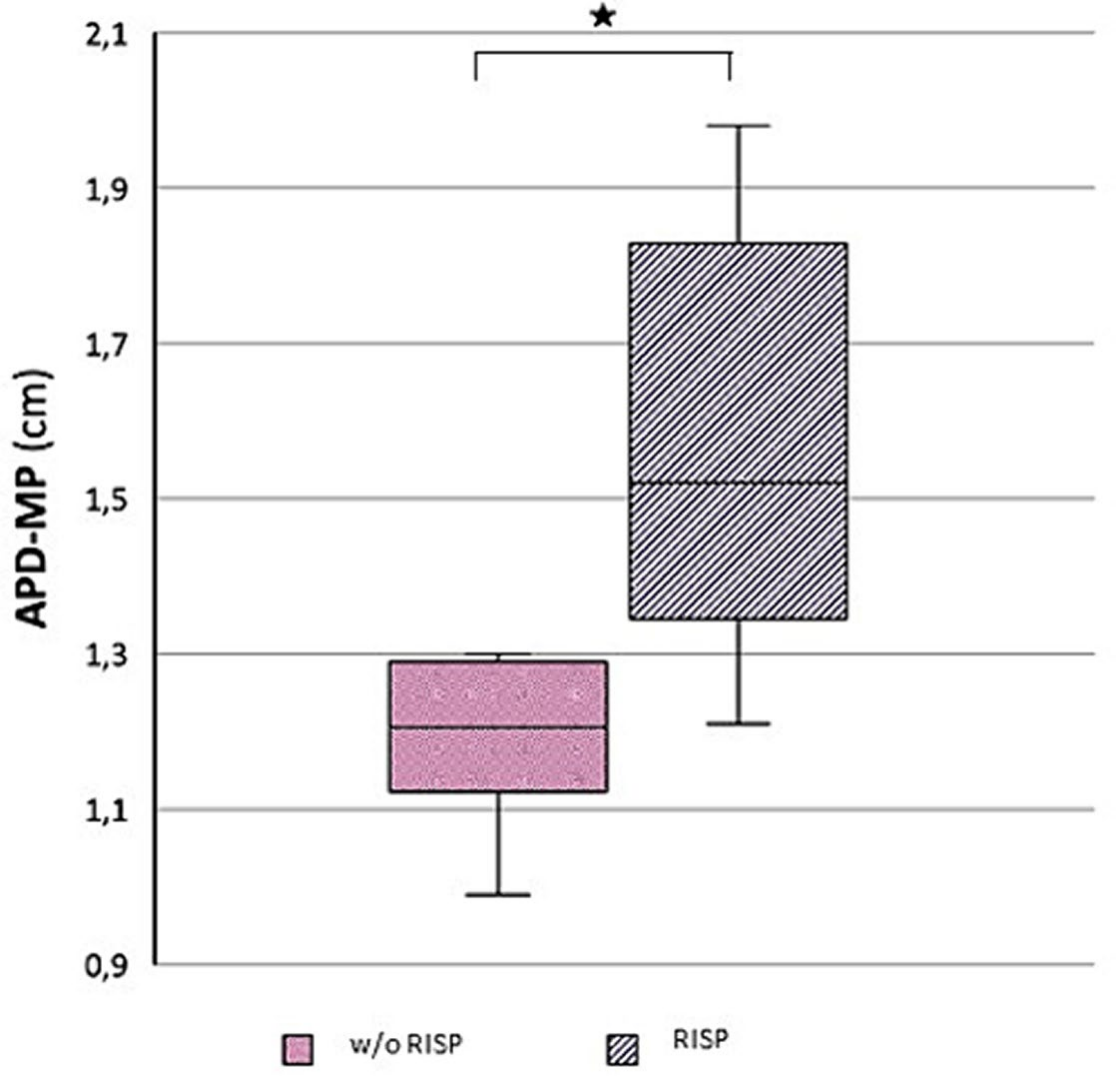
Antero-posterior diameter of mid pons (APD-MP) in w//o RISP (pink) compared to RISP patients (gray).

Morphological examination of particular brain structures revealed statistically significant difference in cerebellar vermis height (H-V), which was statistically significantly greater in patients with RISP compared to healthy individuals (*p* = 0.044, Effect size d = 1.18) (Figure 2). Furthermore, we found statistically significant increase in antero-posterior diameter of midbrain-pons junction in patients with RISP compared to w/o RISP (*p* = 0.018, Effect size d = 0.78) (Figure 3). The more detailed results can be found in Table 2.

**Table 2:** The 2-D MRI measurements of cerebellum, pons and thalamus.

	RISP patients Mean in cm (SD)	w/o RISP Mean in cm (SD)	*P* value
H-V	4.95 (0.45)	4.52 (0.36)	0.044
APD-V	3.18 (0.52)	2.87 (0.55)	0.155
APD-MP	1.56 (0.26)	1.33 (0.45)	0.018
APD-P	2.32 (0.15)	2.22 (0.14)	0.197
APD-LT	3.44 (0.23)	3.62 (0.25)	0.076
TD-LT	1.92 (0.16)	2.00 (0.14)	0.197
APD-RT	3.43 (0.25)	3.66 (0.30)	0.091
TD-RT	1.94 (0.14)	2.02 (0.19)	0.476

H-V, Height of cerebellar vermis; APD-V, Antero-posterior diameter of vermis cerebelli; APD-MP, Antero-posterior diameter of midbrain-pons junction; APD-P, Antero-posterior diameter of mid-pons; APD-LT, Antero-posterior diameter of left thalamus; TD-LT, Transversal diameter of left thalamus; APD-RT, Antero-posterior diameter of right thalamus; TD-RT, Transversal diameter of right thalamus.

## 4 Discussion

### 4.1 Objective macrostructural sleep characteristics

According to our measurements, macrostructural sleep parameters were similar in RISP patients and control subjects. All of the results were within the normal limits.

Sleep paralysis is often linked to some subjective sleep symptoms, such as lower sleep quality observed by the patients (Denis et al., 2018). RISP patients often experience insomnia symptoms (Denis and Poerio, 2017). Possible genetic overlap between sleep paralysis and lowered sleep quality, potentially related to the dysfunction of some clock genes, such as PER 2 gene, has been previously considered (Denis, 2018). Our data do not objectively present any lowered sleep efficiency in neither RISP patients nor control subjects.

Objective polysomnographic measurements on RISP patients have not been widely done. Studies however indicate, that there is not an objective difference in macrostructural sleep parameters between RISP patients and control individuals. As lowered sleep efficiency is comorbid to many sleep disorders, such as sleep apnea and/or periodic leg movements in sleep, it is also possible that the occurrence of these symptoms could increase the chance of RISP manifestation. The relationship between sleep apnea and RISP has not been established yet. Hsieh et al. (2010) did not find any increase in incidence of RISP in patients with OSA compared to those without OSA (Hsieh et al., 2010). This suggests that the presence of OSA did not lead to RISP, even though that might lead to disturbed sleep quality and continuity; another co-factor that is related to RISP (Hsieh et al., 2010). We did not find any clinical symptoms of other sleep disorders in both our patients and controls, such as sleep apnea, periodic leg movement disorder or else, that could interfere with their sleep. Our results therefore suggest, that the objective presence of such sleep disturbance is not necessary for RISP to manifest.

Furthermore, we did not find any macrostructural differences in sleep between the groups. This is also in line with previous research. The study of Klikova et al. (2021) did not find any macrostructural changes in REM sleep in RISP patients (Klikova et al., 2021). However, in terms of microstructural characteristics, they found statistically significant increase in beta activity bi-frontally during REM sleep in RISP patients compared to controls. This could point to some underlaying regulatory processes that are impaired in RISP patients, potentially biochemical in nature. It could be argued that such impairment is reflected in the structure of neuronal network itself, including midbrain and thalamus and potentially other structures such as cerebellum and thus could manifest in size differences of these particular brain structures. Looking into this phenomenon was also the main aim of our work.

### 4.2 Neuroimaging

In order to analyze our data, we firstly looked into the average sizes of cerebellum, midbrain and thalamus, which have already been studied. Cerebellar diameter has been measured in fetus in order to see the developmental characteristics as well as its relationship to gestation age (Vinkesteijn et al., 2000; Mishra et al., 2020). Jandeaux et al. (2019) measured the parameters of cerebellum and pons in children. The authors determine the equations valid in children that provide the average normal size of certain structures given age of the child. Further research also suggests, that diameter of many cerebellar structures including cerebellum and pontine regions changes with age not only in childhood but also in adulthood and correlates with aging (Oguro et al., 1998). Normative data of cerebellum and midbrain regions diameters were recently measured by Metwally et al. (2021), who looked at MRI scans of healthy individuals to determine the referential sizes of particular structures with respect to age (Metwally et al., 2021). We compared our data to the referential limits set by these authors. Our control subjects’ mean values in each of the chosen measurements (H-V, APD – V, APD – MP, APD – P) were within these limits, whereas the RISP individuals seem to exceed them (statistical analysis was not done). The values we observed in healthy individual for each of the measured structures are thus consistent with referential diameters measured in individuals of similar age group (between 18–40 years of age) as defined in previous research (Metwally et al., 2021).

There is a lot of research in which thalamus, alongside other cerebral structures, is measured in volume in both children and adults (Tutunji et al., 2018; Tullo et al., 2019). Less evidence is available in 2-dimensionla measurements. Recent study done by Sajeev et al. (2023) provided 2-dimensional diameters of thalamus obtained from MRI scans (Sajeev et al., 2023). We reached similar mean values as determined by Sajeev et al. (2023). Statistical analysis was not done in this case to further determine whether or not our data differ from the referential values stated by the authors.

To our knowledge, a sporadic evidence has been provided about size of particular brain structures with respect to gender. It is true that our experimental and control groups are matched in age but not in gender. Gender is known to affect size of numerous organs within the human body, including some of the brains structures, such as cerebellum (Giedd et al., 2012). Furthermore, it is generally expected to reach greater sizes in males rather than females. Giedd et al. (2012) found cerebellum to be about 10–13% larger in male compared to female. Our data points out enlargement of H-V and APD-MP in RISP (8 female, 2 male) compared to w/o RISP (3 female, 7 male). If the difference in size was only based on gender, we would expect the RISP individuals to reach smaller diameters compared to the individuals w/o RISP simply because of greater representation of female in the sample. This was not the case in our study, when RISP manifested greater mean diameters compared to w/o RISP. Even though the difference in gender representation in RISP and w/o RISP group could have partially altered the results, we argue that the direction of this alteration would probably decrease the actual mean difference in size and thus lower the chance to find statistically significant result. As we found our results to be significantly different with adequate effect size, we argue that gender matched sample would even increase the power of our findings.

### 4.3 Potential role of cerebellum, midbrain, pons and thalamus in RISP

There is not a large amount of literature available exploring the morphological changes in the brain of RISP patients. We pointed our research toward the structures that are known to play a role in sleep regulation, such as thalamus, a key player in transition into deeper sleep stages but also controlling for sleep–wake regulation (Gent et al., 2018).

#### 4.3.1 Cerebellum and pons

Current research suggests that cerebellum plays an important role in the control of REM sleep and its transition to wakefulness and thus might contribute to the development and manifestation of REM parasomnias. During REM sleep, cerebellum shows increased neuronal activity compared to wakefulness and seems to contribute to the maintaining of muscle atonia during REM sleep (Hong et al., 2009; Miyauchi et al., 2009). In addition, cerebellum regulates autonomic inputs from the amygdala, periaqueductal gray (PAG), and thalamus and expresses parasympathetic and sympathetic outputs to the brainstem ventilatory and oculomotor neurons during REM sleep (Dharani, 2005). Cerebellar cortex also seems to collaborate with activity of Purkinje cells (PC) during REM sleep and its transition to wakefulness and thus contributes to signal that terminates REM sleep and leads the transition to wake (Zhang et al., 2020).

Morphological changes in cerebellum, as well as changes in connectivity of cerebellum, were already described in other REM parasomnias, such as REM sleep behavioral disorder (RBD) (Liu et al., 2021). In idiopathic RBD patients an increased gray matter volume was found in cerebellum compared to healthy control subjects (Chen et al., 2022). The authors speculate the compensatory role of this phenomenon. Contrary to these findings, Lee et al. (2023) found decrease of cerebellar volume in iRBD patients when compared to healthy controls (Lee et al., 2023). Authors however worked with individuals with mean age above 60 years. Other research provides evidence of some degree of reduction in volume of many brain structures with age even in healthy population (Metwally et al., 2021). It is possible that the deterioration in size of some brain structures is more prominent in individuals with iRBD than in healthy control subjects, leading to a greater decrease in volume, as found by Lee et al. (2023). To our knowledge, no such research was done in RISP individuals.

Our data show increased length of cerebellar vermis, which could be similar case of compensatory mechanism to otherwise ineffective or partial effective regulatory mechanisms, similar to what Chen et al. (2022) described in idiopathic RBD patients. As cerebellum was found to increase its activity during REM sleep and to play a role in maintaining muscle atonia, the above-described morphological changes could be a morphological marker of reduced effectivity of these mechanisms that might eventually contribute to the occurrence of RISP episodes. As we found statistically significant increase in some of cerebellar diameters it could be argued that also the total volume of cerebellum might increase in RISP compared to healthy population, which would be consistent with findings of Chen et al. (2022) when applied on REM sleep parasomnias all together.

Pontine regions are traditionally related to the REM sleep regulation and maintaining REM sleep atonia (Park and Weber, 2020; Vetrivelan and Bandaru, 2023). Studies suggest that the destruction of various pontine regions, such as by a growing tumor in the pontine areas, may lead to the development of narcolepsy accompanied by sleep paralysis and hypnagogic hallucinations (Stahl et al., 1980; Pr et al., 2022). Pathological processes in pontine regions, such as demyelinating lesions in multiple sclerosis could also lead to the development of narcolepsy, RBD and/or even sleep paralysis (Foschi et al., 2019).

Above all, sublaterodorsal nucleus (SLD), especially its glutamatergic portion (Vetrivelan and Bandaru, 2023) seem to play a key role in keeping muscle atonia during REM sleep, which was found in many studies (Torontali et al., 2019). Thus, SLD inevitably plays role also in manifestation of sleep paralysis; the muscular paralysis after artificial stimulation of SLD was already proved in experiment (Torontali et al., 2014). Chemical stimulation might have various effects; whereas cholinergic modulation of SLD might lead to REM sleep generation, the GABAergic and glutamatergic influence affect REM sleep control (Lu et al., 2006). Cholinergic regulation of SLD also profoundly affects muscle paralysis (Kayama and Koyama, 2003).

Our data might suggest, that the increased diameter of midbrain-pons junction region in RISP could be a similar compensatory mechanism as in case of increased cerebellar vermis diameter.

Due to technical specifications of our method we are not able to refer to whether the enlargement is caused by the increased volume of gray matter and/or white matter. Previous studies have focused mainly on the gray matter. Previous studies done in iRBD also reported the decreased in volume in some cortical regions (Chen et al., 2022). This was not explored by our study and we therefore cannot comment on it.

#### 4.3.2 Thalamus

It is well known that switching between REM and NREM sleep is controlled by interactions of cholinergic and adrenergic neurons in brain stem (Pace-Schott and Hobson, 2002). Thalamus, however, plays a role in REM sleep, too. Simor et al. (2021) found the activity of specifically anterior nuclei of the thalamus differs between phasic and tonic REM sleep and also thalamocortical activity differs according to the phase of REM sleep (Simor et al., 2021). Thalamic medial pulvinar was found to express slow waves in REM sleep and overall thalamic activation during REM sleep (Szabo et al., 2022). The specific activity of thalamic nuclei was also associated with eye movements during REM sleep, suggesting the relationship between thalamus and motor control networks in REM sleep (Kempf et al., 2009).

We argue that as there is an evidence of the role of the thalamus in motor activity during REM sleep, the morphological and/or chemical changes that occur in the thalamus could have an impact on the occurrence of sleep paralysis events. Our 2-dimensional measurements did not find any statistically significant differences in the antero-posterior diameter of thalamus nor transversal diameter bilaterally. This could mean, that thalamus is not involved in the development of sleep paralysis events. However, the fact, that the size does not change in RISP patients does not fully exclude potentially underlying chemical processes that affect REM sleep regulations that could contribute to the development and manifestation of RISP.

Yet the fact that thalamus was of a normal size in both patients with and w/o RISP could further explain why we did not find any macrostructural changes in sleep of our participants.

### 4.4 Potential clinical implications

In order to use these measurements in clinical practice we would firstly need to determine the cut-off in which the diameter exceeds normal limits and becomes a marker of RISP. This would certainly require replication of our data on larger sample size. It can be assumed that patients with RISP would also show typical clinical symptoms, and therefore the measurement of selected brain structures would serve more as a confirmation of the diagnosis rather than a diagnostic tool. There is a possibility, that even enlarged, the diameter of these structures (H-V, APD – V, APD – MP, APD – P, APD-LT, TD – LT, APD – RT, TD – RT) changes with respect to the severity of RISP, measured by the number of RISP episodes over given amount of time. However, clinically we see large inter-individual differences between individual patients in terms of frequency of the events. In order to bring this into practice, it would be necessary to set up a specific protocol of video-polysomnography examination and follow-up MRI and thus to be able to associate neuroanatomical findings with the exact and current clinical manifestation of RISP. If this is successful, then the measurement of these selected brain structures could be used to categorize the severity of RISP.

Furthermore, our conclusions could be used in prevention. The measurement itself can be performed very easily in routine MRI imaging. Therefore, if we were to measure these structures, for example, in patients with pre-existing neurological or psychiatric problems and detect specific enlargement, we could anticipate changes in REM sleep (for example, in the sense of RISP) and respond in time with appropriate clinical intervention, such as by education in the field of sleep hygiene or with polysomnography so as to prevent the possible progression of the patient’s condition and the experiences of fear and anxiety that usually accompany episodes of RISP. All this could in fact help patients to overcome the situation and spare them from negative experiences.

## 5 Limitations

In order to take these findings closer to clinical applications, we would certainly need to increase our sample size to see if the results could be replicated. Even though the sample size is limited, the control subjects mean diameters of each of the measured values (H-V, APD – V, APD – MP, APD – P, APD-LT, TD – LT, APD – RT, TD – RT) were consistent with previous research using similar strategy (measurement was done by using 2-dimensional MRI scans and similar age groups was determined), which could add some value to our findings. Furthermore, the longitudinal component would be needed in order to assess the potential relationship of the size of cerebellum and midbrain and the frequency of RISP episodes in time. Providing measurement of volume could also bring more insight. Finally, our technology did not provide adequate precision to be able to measure the sizes of certain thalamic or pontine nuclei, which could also have been helpful in understanding the pathophysiology of RISP.

## 6 Conclusion

Our findings suggest increased diameter of particular brain structures, namely cranio-caudal length of vermis cerebelli and antero-posterior diameter of midbain-pons junction in RISP patients compared to w/o RISP subjects. As both structures are clearly related to the regulation of REM sleep and the transition between REM sleep and wakefulness, we assume this increase in size to be a feature of RISP. Our study does not provide adequate data to prove the specific function of this enlargement. We can speculate this difference to be a type of over-compensatory mechanism to the otherwise inadequate function of signaling pathways involved in REM sleep regulation and transition between REM sleep and wakefulness.

## Data availability statement

The raw data supporting the conclusions of this article will be made available by the authors, without undue reservation.

## Ethics statement

The studies involving humans were approved by Research ethic committee of the National Institute of Mental Health. The studies were conducted in accordance with the local legislation and institutional requirements. The participants provided their written informed consent to participate in this study.

## Author contributions

EM: Writing – review, editing, Writing – original draft, Visualization, Project administration, Methodology, Investigation, Formal analysis, Data curation, Conceptualization. MK: Writing – review, editing, Resources, Investigation, Funding acquisition. AD: Writing – review, editing, Investigation, Formal analysis, Data curation. JB: Writing – review, editing, Writing – original draft, Supervision, Methodology, Conceptualization.
